# Cerebrovascular and Neuroprotective Effects of Adamantane Derivative

**DOI:** 10.1155/2014/586501

**Published:** 2014-03-20

**Authors:** Ruben S. Mirzoyan, Tamara S. Gan'shina, Denis V. Maslennikov, Georgy I. Kovalev, Ivan A. Zimin, Boris M. Pyatin, Nina I. Avdyunina, Anna M. Kukhtarova, Nelly G. Khostikyan, Vahe S. Meliksetyan, Cristina B. Alikhanyan, Narine R. Mirzoyan

**Affiliations:** ^1^Zakusov Institute of Pharmacology RAMS, 8 Baltiyskaya Street, Moscow 125315, Russia; ^2^Yerevan State Medical University after Mkhitar Heratsi, 2 Koryun Street, 0025 Yerevan, Armenia

## Abstract

*Objectives*. The influence of 5-hydroxyadamantane-2-on was studied on the rats' brain blood flow and on morphological state of brain tissue under the condition of brain ischemia. The interaction of the substance with NMDA receptors was also studied. *Methods*. Study has been implemented using the methods of local blood flow registration by laser flowmeter, [^3^H]-MK-801binding, and morphological examination of the brain tissue. We used the models of global transient ischemia of the brain, occlusion of middle cerebral artery, and hypergravity ischemia of the brain. *Results*. Unlike memantine, antagonist of glutamatergic receptors, the 5-hydroxyadamantane-2-on does not block NMDA receptors but enhances the cerebral blood flow of rats with brain ischemia. This effect is eliminated by bicuculline. Under conditions of permanent occlusion of middle cerebral artery, 5-hydroxyadamantane-2-on has recovered compensatory regeneration in neural cells, axons, and glial cells, and the number of microcirculatory vessels was increased. 5-Hydroxyadamantane-2-on was increasing the survival rate of animals with hypergravity ischemia. *Conclusions*. 5-Hydroxyadamantane-2-on, an adamantane derivative, which is not NMDA receptors antagonist, demonstrates significant cerebrovascular and neuroprotective activity in conditions of brain ischemia. Presumably, the GABA-ergic system of brain vessels is involved in mechanisms of cerebrovascular and neuroprotective activity of 5-hydroxyadamantane-2-on.

## 1. Introduction

The brain ischemia is accompanied by a cascade of pathophysiological and biochemical processes caused by oxygen and energy deficiency as well as functional changes like failure of balance between excitatory and inhibitory processes in CNS, which leads to irreversible damage of the neural tissue. The decrease in the rate of aerobic glycolysis brings, to intracellular acidosis, decrease in work efficiency of the sodium-potassium pump, changes in ionic gradients, enhanced discharge of excitatory amino acid glutamate, high concentrations of which cause increased flow of calcium ions into the cell, and activation of enzyme systems. Under these conditions the process of oxidative phosphorylation fails and toxins and free radicals, including NO, are produced; lipid peroxidation is activated and oxidative stress develops. All this results in damage of cytoplasmic organelles' membrane proteins and molecules of DNA and RNA, while expression of immediate early genes triggers the mechanism of cell death through necrosis or apoptosis [1–4].

It should be noted that GABA system is of high interest in conditions of brain ischemia, as GABA-ergic mechanisms play important role in terms of removing the imbalance between excitatory and inhibitory systems in CNS. It was demonstrated that concentration of GABA increases in early stages of ischemia and the high concentration of agonist usually leads to decreased sensitivity of receptors through negative feedback mechanism [5, 6]. There are also literature data stating that compounds, enhancing GABA-ergic transmission, have neuroprotective activity. These are agonists and modulators of GABA_A_ receptors, inhibitors of GABA transaminase, and GABA reuptake inhibitors [6]. GABA system, along with participation in inhibitory processes, plays important role in regulation of cerebral vascular tone. It is known that brain vessels contain GABA, as well as enzymes synthesizing and metabolizing GABA [7–9]. In pial vessels a high affinity of binding with GABA_A_ receptors was observed at muscimol, a GABA_A_ receptor agonist [10]. Earlier we showed that substances with GABA-ergic mechanism of action cause selective improvement of cerebral blood flow in conditions of ischemic damage of the brain [11–15].

Our attention was attracted by adamantane derivatives, which are able to block NMDA receptors and are widely used in neurological practice. Amantadine and memantine are used in the treatment of neurodegenerative diseases. Memantine in experiments prevents the development of neurotoxicity provoked by activation of NMDA receptors. It also blocks N-cholinoreceptors. Memantine improves cognitive functions in patients with mild to moderate vascular insufficiency [16, 17]. An adamantane derivative, 5-hydroxyadamantane-2-on, has ability to improve the blood supply of lower extremities in patients with chronic occlusive (obliterative) arterial disease of lower extremities [18].

In compliance with this, the aim of the current study was the comparative investigation of effects of 5-hydroxyadamantane-2-on and memantine on cerebral blood flow of intact rats and rats exposed to global transient brain ischemia. The study aimed also to investigate neuroprotective activity of 5-hydroxyadamantane-2-on in conditions of local permanent brain ischemia caused by occlusion of middle cerebral artery, with evaluation of morphological state of brain tissue. The survival rate of rats with hypergravity ischemia also was the objective of the study. There are some materials included in current study that analyze the mechanism of cerebrovascular and neuroprotective activity of studied substance.

## 2. Materials and Methods

Experiments of studying the brain blood flow were conducted on 76 narcotized (urethane 1.2 g/kg, i/p) nonlinear male rats weighting 180–400 g. Experiments of middle cerebral artery occlusion were conducted on 28 nonlinear male rats weighting 250–300 g, narcotized with chloral hydrate (400 mg/kg, i/p). 50 wakeful rats were involved in experiments with hypergravity ischemia and brains of 6 male Wistar rats (200–250 g) were used in experiments of radioligand binding.

The state of cerebral blood circulation in animals was estimated by the method of laser Doppler flowmetry. To register the local cerebral blood flow in parietal lobe of the brain cortex, BIOPAC MP100 system (USA) with laser Doppler flow module LDF100C was used; LD channel was calibrated in TPU (tissue perfusion unit) because this unit has better (~1) proportion to mL/min/100 grams of tissue than BPU (default unit for LDF100C). The 08 mm diameter needle-shaped detector of flowmeter was placed on the parietal lobe of the brain cortex of rats using micromanipulator and balancing arm. Changes of arterial pressure were registered simultaneously through a polyethylene catheter, preliminary inserted into the femoral artery. Based on data of detectors of blood flow and pressure, vascular resistance was estimated in real time. The recording of characteristics of blood flow, arterial pressure, and vascular resistance was conducted on polygraph “BIOPAC” (USA), connected with a personal computer. Investigating substances were administered through the polyethylene catheter into the femoral vein of animals.

The global transient ischemia in rats was achieved by 10-minute occlusion of both common carotid arteries with simultaneous decrease of arterial pressure down to 40–50 mm Hg, using method of bloodletting and following reperfusion.

The local permanent ischemia was obtained by the method of Tamura et al. [19], modified by Topchian et al. [20].

The morphological state of brain tissue was assessed on 6th and 12th days after occlusion of middle cerebral artery. These intervals were chosen based on existing literature data, according to which the most profound structural changes in the brain after occlusion of middle cerebral artery in rats are seen on 6th and 12th days [20, 21]. Animals were decapitated on 6th or 12th day after operation. The brain was extracted and fixed in 10% neutral solution of formaldehyde during the preparation for morphological examination and then sagittal incision was done including tissue supplied by middle cerebral artery. Furthermore, the tissue was fixed in a paraffin block. This block was sliced step by step into tissue specimens and was stained with universal histological dye, hematoxylin/eosin.

The first two series of morphological examination were done on two control groups of animals which underwent an occlusion of middle cerebral artery and were decapitated after 6 and 12 days, respectively.

In the following series of experiments animals were involved with occlusion of middle cerebral artery and treated with 5-hydroxyadamantane-2-on (100 mg/kg, i/p, once daily) 30 minutes after the occlusion and the following 6 and 12 days, respectively.

To study the survival rate of animals in conditions of hypergravity loading [22] wakeful rats were placed into special containers of centrifuge in craniocaudal orientation relatively to the vector of acceleration. In case of craniocaudal vector of acceleration (9 g during 12 minutes) the movement of blood occurs towards caudal orientation: as a result, the perfusion pressure falls dramatically down to the zero in all vessels of the head and brain ischemia develops.

### 2.1. Binding to NMDA Receptors

Binding to NMDA receptor subtype was studied using the method [23] with modifications. Tritium-labeled MK-801 (dizocilpine) with a specific activity of 210 Ci/mol was used [24]. The rat's hippocampal tissue was homogenized in a Potter homogenizer (Teflon glass) in 10 volumes of 5 mM HEPES/4.5 mM Tris buffer (pH 7.6) containing 0.32 M sucrose (buffer-1). The homogenate was diluted with 50 volumes of buffer-2 (5 mM HEPES/4.5 mM Tris buffer, pH 7.6) and centrifuged for 10 min at 1000 g. The supernatant was taken and again centrifuged for 20 min at 25000 g. The pellet was homogenized in 50 volumes of buffer-2 and centrifuged for 20 min at 8000 g. The supernatant and its soft unstable upper coat were taken and centrifuged for 20 min at 25000 g. The resulting precipitate was suspended in buffer-3 (5 mM HEPES/4.5 mM Tris buffer containing 1 mM NaEDTA, pH 7.6), and the suspension was centrifuged again. This washing procedure was repeated four times, with EDTA being excluded in the last wash. The final precipitate was resuspended in 5 volumes of buffer-2 and then stored in liquid nitrogen. The reaction mixture (final volume 0.5 mL) contained 200 *μ*L of buffer-2, 50 *μ*L of labeled ligand (50 nM solution), and 250 *μ*L of protein suspension. The nonspecific binding was determined at the presence of 50 *μ*L of unlabeled ligand. The reaction mixture was incubated at room temperature (20°C) for 2 h. After the incubation, the samples were filtered through GF/B filters (Whatman) which were presoaked in 0.3% polyethylenimine for 2 h at 4°C. Each test tube was washed 1 time with cold buffer-2, and the filters were then washed three times with the same buffer volume. The filters were dried on air and transferred to the scintillation vials. The radioactivity was determined on a Wallac 1411 scintillation counter with a counting efficiency of approximately 45%.

## 3. Statistical Analyses

The statistical analysis of data was carried out using the software Statistica 8.0 (Statistica Inc., USA). The normal distribution was defined by Shapiro-Wilk test. Generally, normal distribution was lacking; thus for the following analysis nonparametrical method of Wilcoxon signed-rank test was used. Survival data of animals in conditions of circulatory ischemia was calculated by Fisher criterion.* GraphPad Prism 5* software was used in experiments of radioligand binding. Results were considered statistically significant, when *P* < 0.05.

## 4. Results 

### 4.1. The Influence of Adamantane Derivatives, 5-Hydroxyadamantane-2-on and Memantine, on Local Blood Flow of Brain Cortex in Intact Rats and Those with Global Transient Ischemia

**Figure 1:**
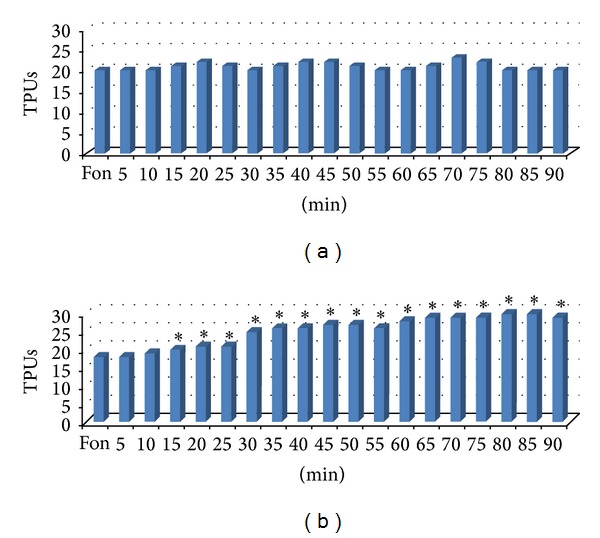
The influence of 5-hydroxyadamantane-2-on (100 mg/kg, IV) on CBF of intact rats (a) and animals with global transient brain ischemia (b).

Experiments on intact rats revealed that 5-hydroxyadamantane-2-on with intravenous administration of dose 100 mg/kg does not cause significant changes in blood flow of rats' brain cortex (Figure 1(a)). The adamantane derivative causes decrease of blood pressure in intact rats from 30 to 90 minutes by average of 11–14%.

In 10 minutes after intravenous administration of memantine (5 mg/kg) to intact narcotized rats, the decrease of local cerebral blood flow by 15% in average was registered. Further decrease of blood flow was observed, which reached 53% by the end of experiment. Memantine virtually did not affect the level of arterial blood pressure.

**Figure 2:**
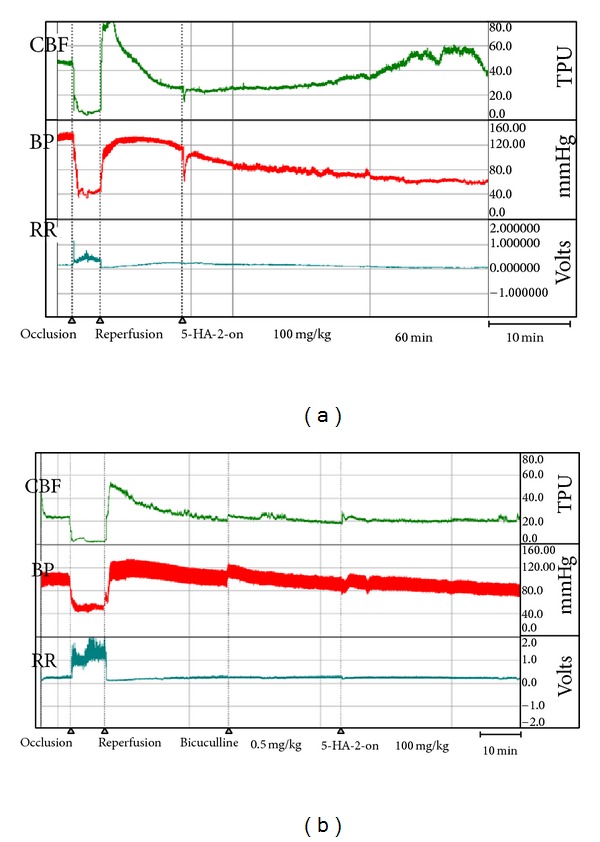
The influence of 5-hydroxyadamantane-2-on (100 mg/kg, IV) (5HA-2-on) on local cerebral blood (CBF), blood pressure (BP), and rated resistance (RR) of rats after global transient ischemia of the brain (a) and with action of bicuculline (b).

On the next series of experiments the influence of adamantane derivatives was studied on the brain blood flow after global transient ischemia. It was shown that 5-hydroxyadamantane-2-on (100 mg/kg, IV) causes slowly developing increase of local cerebral blood flow, which reaches its peak after 60 minutes (76.5%). This improvement of cerebral blood flow over the initial level lasts till the end of experiment (90 minutes and more) (Figures 1(b) and 2(b)). In these conditions, adamantane derivative lowers arterial pressure in rats by 11% in average.

Memantine (5 mg/kg) decreased (though in a less degree) local cerebral blood flow in the brain cortex of experimental animals as well as of intact rats. In the same experiment with memantine, arterial blood pressure failed statistically significantly by 14% in average.

Thus, the conducted experiments established that cerebrovascular effect of 5-hydroxyadamantane-2-on, unlike memantine, develops in conditions of ischemic brain damage and is absent in intact animals. It should be noted that increase of blood flow is a result of the influence of the substance on brain vascular tone. The results of rated resistance prove this, as adamantane derivatives along with increase in brain blood flow cause decrease in arterial pressure.

### 4.2. The Investigation of Neurochemical Mechanisms of Action of 5-Hydroxyadamantane-2-on

It is known that amino derivatives of adamantane (amantadine, memantine) possess their protective effects via noncompetitive antagonism with glutamate for NMDA receptors. However, there is no evidence of such mechanism for hydroxy derivatives, particularly 5-hydroxyadamantane-2-on. Thus, we observed the influence of this substance on binding of ligand of receptor's channel site, [^3^H]-MK-801 (dizocilpine), with hippocampal membranes of rat. This effect was compared with that of memantine (Figure 6).

Results suggest that memantine actively competes with labeled dizocilpine in size of IC_50_ = 1.14 *μ*M (95% confidence intervals: 0.82–1.57 *μ*M,* GraphPad Prism 5*), while 5-hydroxyadamantane-2-on has no effect through the all range of concentrations.

In further studies we observed the influence of 5-hydroxyadamantane-2-on on local brain blood flow while blocking with bicuculline, a GABA_A_ receptor blocker. This was reasonable, as substances with GABA-ergic mechanism of action reveal vasodilating activity in ischemic conditions. Bicuculline was administered by dose 0.5 mg/kg after ischemic damage of the brain, and then adamantane derivative was administered after 30 minutes. It was found out that, while blocking of GABA-receptors with bicuculline, 5-hydroxyadamantane-2-on does not increase the local brain blood flow (Figure 2(b)). These results indicate the participation of GABA-ergic mechanisms of cerebrovascular effects of 5-hydroxyadamantane-2-on in brain ischemia.

### 4.3. The Influence of 5-Hydroxyadamantane-2-on on Morphological State of Brain Tissue after the Occlusion of Middle Cerebral Artery

To study the neuroprotective activity of 5-hydroxyadamantane-2-on, its influence was observed on morphological injury of brain tissue provoked by occlusion of middle cerebral artery.

**Figure 3:**
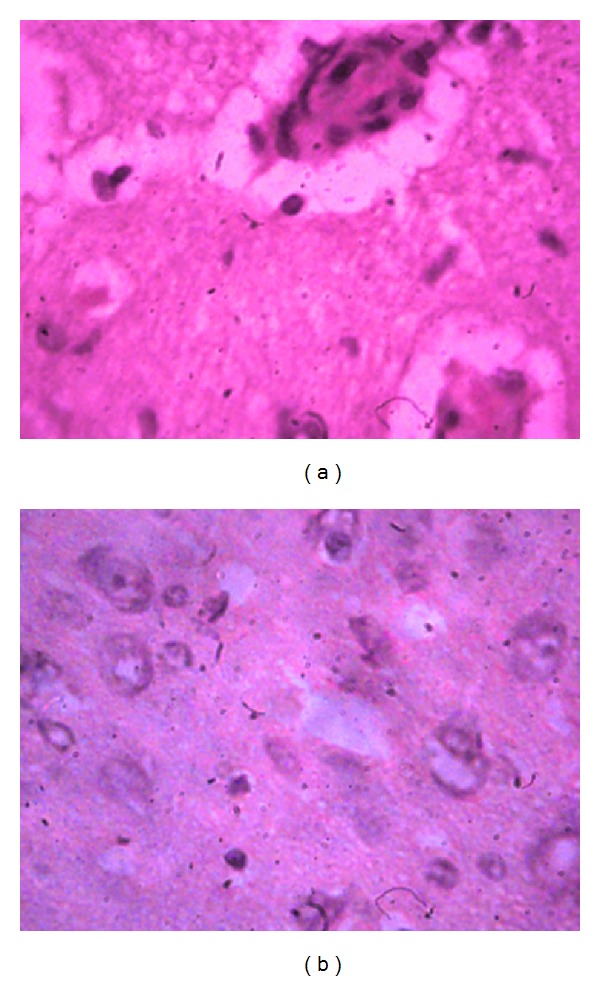
Morphological picture of brain tissue of rats with occlusion of middle cerebral artery. (a) An expressed perivascular and pericellular edema of the brain, stasis in arterioles, and foci of empty neural and glial cells (staining hematoxylin/eosin ×400). (b) Against the background of necrobiosis, anuclear shadow cells of neurons as well as dystrophic neural and glial cells are seen (staining hematoxylin/eosin ×400).

The morphological examination of specimens of control group (with occlusion of middle cerebral artery and without treatment) revealed an expressed perivascular and pericellular edema of brain tissue. In edematous brain tissue there are visible cells with dystrophy of neuronal cytoplasm and nucleus, zones of karyorrhexis, karyopyknosis, and karyolysis of neural and glial cells. Capillars and arterioles in state of stasis and microthrombosis are seen along with empty microcirculatory vessels. These arterioles are surrounded by emptied neural and glial cells (Figure 3(a)). There are zones of necrobiosis through all layers of cortical cells with wash-out, lysis of neural and large glial cells' nucleoplasm. Anuclear, necrotizing shadow cells as well as areas of dystrophic neurosecretory cells of paraventricular and supraoptic nuclei are detectible against the background of necrobiosis of cortical pyramidal cells (Figure 3(b)). Borders of all cortical layers are cleared. Several hypertrophic neurons with hyperchromic nuclei are seen between or near the profound ischemic foci. This indicates the intracellular regeneration of individual intact neural and glial cells. There are also proliferation zones of oligodendrocytes and astrocytes. Similar changes are registered also in neurosecretory cells located in the basin of middle cerebral artery. Punctuate and linear hemorrhages, meningeal edema, stasis in vessels of pia, and dura mater are seen in intercellular substance. Zones of hemosiderosis are also visible.

These obtained results correspond to the literature data of previous studies that showed up bilateral disturbances of brain blood flow, hypoxic damage of cortical neurocytes, and conduction pathways synaptic terminals in analogous periods after occlusion of middle cerebral artery.

In the next two series the influence of 5-hydroxiadamantane-2-on (100 mg/kg, i/p) was studied on morphological state of brain tissue of rats with occlusion of middle cerebral artery after 6 (3rd group) and 12 (4th group) days of treatment.

The morphological changes of brain tissue were examined on sagittal incisions of brain. This made it possible to detect tissue changes in regions of both left and right middle cerebral arteries and to provide comparative morphological picture of occluded (left) and not occluded (right) hemispheres.

Some neurons and groups of neurons are seen in the state of necrobiosis on serial tissue specimens of the 3rd group of animals, in the left hemisphere. Small foci of empty neural and glial cells and dystrophic changes of pyramidal cells of brain cortex are also seen. Pericellular and perivascular edema is marked slightly, vessels of microcirculatory bed are hyperemic as well as foci of extravasates, and old small hemorrhages with hemosiderin pigment are seen. Foci of glial cells', oligodendrocytes', and astrocytes' proliferation are also found.

Processes of dystrophy and individual cells in conditions of necrosis and necrobiosis are seen in neurosecretory cells of paraventricular and supraoptic nuclei. Against the background of above-described changes large hypertrophic neurosecretory cells are detected in paraventricular and supraoptic nuclei with the presence of multiple neurosecretory granules.

**Figure 4:**
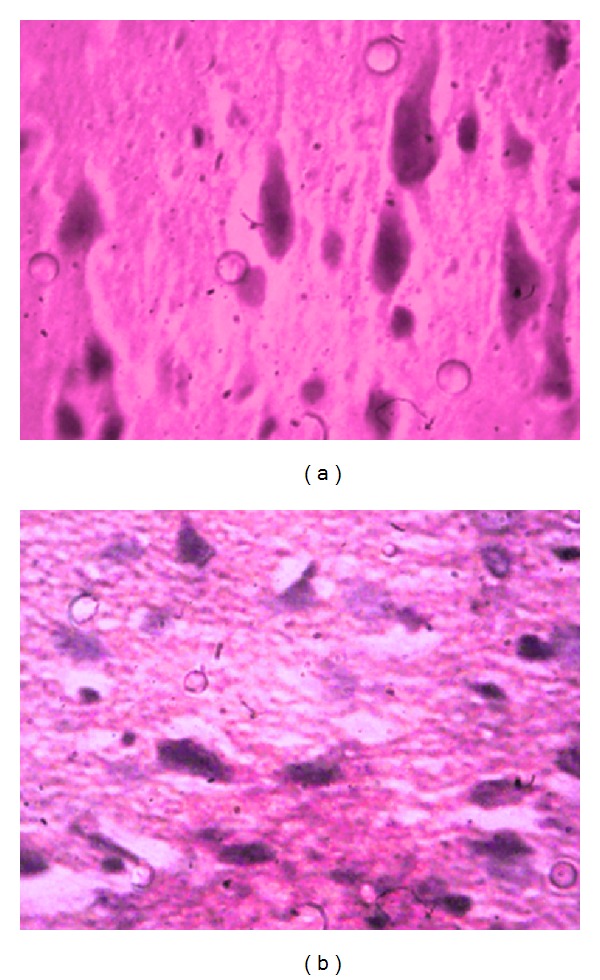
Morphological picture of brain tissue of rats with occlusion of middle cerebral artery after treatment with 5-hydroxyadamantane-2-on. (a) Hypertrophic pyramidal cells with preserved neuritis (staining hematoxylin/eosin ×400); (b) along with hypertrophic cells, those without axons and with wrinkled cytoplasm and nucleus are seen (staining hematoxylin/eosin ×400).

Similar changes and compensatory-regenerative processes are observed in all pyramidal cells of the cortex (Figure 4(a)). Axons of above-mentioned hypertrophic neurons are preserved through all length.

**Figure 5:**
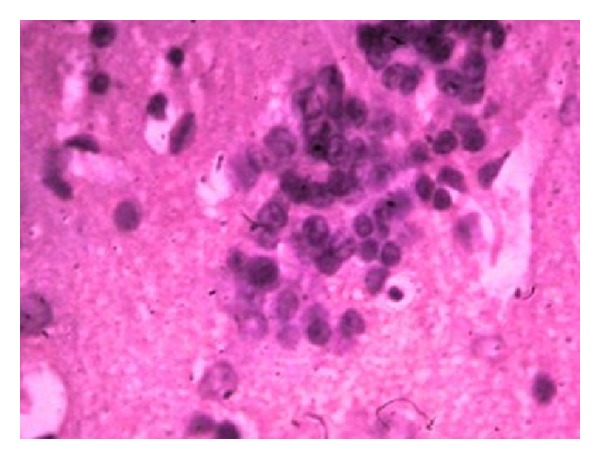
Morphological picture of brain tissue of rats with occlusion of middle cerebral artery after treatment with 5-hydroxyadamantane-2-on. Lymphoid-macrophagal reaction is observed in zones of necrosis and marked edema in response to impaired polycystic changes of brain tissue (staining hematoxylin/eosin ×400).

**Figure 6:**
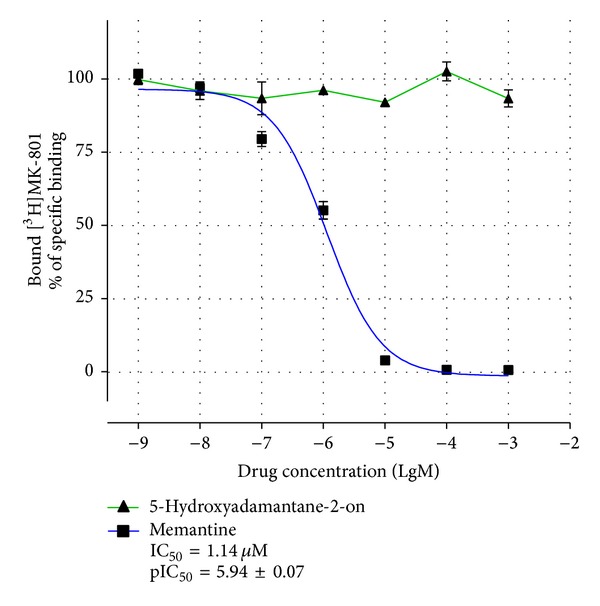
The influence of memantine and 5-hydroxyadamantane-2-on on specific binding of [^3^H]-MK-801 with rat hippocampal membranes (pIC_50_ = m ± S.E.).

There are cells without neurites in the basin of middle cerebral artery, on the left side, with wrinkled cytoplasm and nucleus (Figure 4(b)). In ependymal cells of brain ventricles proliferation is observed, while in sinusoidal vessels hyperemia is observed. In response to harmful, polycystic changes there is a focal lymphoid-macrophagal reaction in individual preserved zones of necrosis and edema (Figure 5). There are also microvessels with obliterating white thrombotic bodies.

In the 4th group of animals the treatment with 5-hydroxiadamantane-2-on was conducted in the course of 12 days after occlusion of middle cerebral artery and the observed morphological changes had more reparative-regenerative and proliferative character. In general, the disposition of layers of cortical cells is preserved. There is a slight pericellular and perivascular edema of meninges and brain tissue along with hyperemia of microcirculatory vessels. Small foci of shadow neural and glial cells are visible. In the basin of left middle cerebral artery there are generally wide zones of pyramidal neurosecretory and glial cells of normal size, with precise borders between cytoplasm and nucleus and with available nucleolus. Granules of neurosecretory substance are observed in neurosecretory cells. There are ischemic zones in certain parts of third ventricle, where contours of neurons, glial cells, neuritis, and neuropile are vague. Hypertrophic neurocytes with hyperchromic nuclei are observed near these zones as well as functionally active neurosecretory cells with multiple secretory granules.

The morphological picture of the right hemisphere in basin of right middle cerebral artery was also examined on the same tissue specimens and a following comparison characteristic of morphological picture was made (the picture of secondary control on the same animals). There are insignificant perivascular and pericellular edema, hyperemia of microcirculatory bed in all series of the experiment (in control groups and groups treated with 5-hydroxyadamantane-2-on for 6 and 12 days), on the right hemisphere. There are compensatory-regenerative processes, that is, intracellular regeneration of organelles, hypertrophy of pyramidal cells and large glial cells, and increase in number of microcirculatory vessels. These processes are more significant on the 12th day after occlusion. Typically, in conditions of damage of one part of the brain, other parts of brain or another hemisphere (right—in this case) compensate and repair morphofunctional balance of the CNS.

### 4.4. The Influence of 5-Hydroxyadamantane-2-on on Survival Rate of Animals in Conditions of Hypergravity Loading

Anti-ischemic properties of adamantane derivative were observed on trials with wakeful rats in conditions of hypergravity loading. 5-Hydroxyadamantane-2-on was studied in doses 50–100–150–200 mg/kg injected intraperitoneally. Experiments revealed that the mortality rate from hypergravity overload is 80% in control group, while survival rate is 20%. After administration of adamantane derivative by the dose 100 mg/kg, survival rate rises up to 80% (Table 1). In doses 150–200 mg/kg survival rate was 60%. In these cases the data are not statistically significant. It can be concluded that adamantane derivative in doses 100 mg/kg reveals anti-ischemic effects in conditions of hypergravity ischemia like its effects in global permanent ischemia.

## 5. Conclusions

This paper introduces new data about cerebrovascular and neuroprotective activities of adamantane derivative, 5-hydroxyadamantane-2-on. Comparative study of cerebrovascular activity was done between two adamantane derivatives: 5-hydroxyadamantane-2-on and memantine, antagonist of glutamate receptors. There was revealed a substantial difference between their effects on blood flow of ischemic brain. 5-Hydroxyadamantane-2-on enhances blood flow and decreases vascular tone of rats with global permanent brain ischemia and has no such cerebrovascular effects on brains of intact rats, while memantine decreases blood flow in both intact and ischemic brains.

Further experiments studied the influence of 5-hydroxyadamantane-2-on on NMDA receptors taking into consideration that many adamantane derivatives, memantine particularly, are able to block NMDA receptors. It was found out that unlike memantine and many other amino derivatives of adamantane, 5-hydroxyadamantane-2-on does not interact with this site of NMDA receptors. Moreover, both of these compounds did not compete with [^3^H]-7OH-DPAT for the binding site of dopamine receptors as well as with [^3^H]-8OH-DPAT and [^3^H]-ketanserin for the binding site of 5-HT1A- and 5-HT2A receptors, respectively.

The investigation of neuroprotective activity of 5-hydroxyadamantane-2-on in conditions of permanent occlusion of middle cerebral artery in rats was the other objective of this study. The compound was administered (100 mg/kg) 30 minutes after occlusion, once daily during 6 and 12 days. Experiments have shown that 5-hydroxyadamantane-2-on promotes the recovery of compensatory regeneration processes in neural cells, axons, glial cells. It also increases the quantity of vessels of microcirculatory bed. Results obtained indicate the neuroprotective effects of compound that was confirmed by results from experiments with hypergravity ischemia in rats, where survival rate of animals was increased by treatment with the same dose of 5-hydroxyadamantane-2-on.

One of neuroprotective mechanisms demonstrated for 5-hydroxyadamantane-2-on was its capacity to enhance the blood flow of ischemic brain, which was not manifested in the background of action of specific GABA_A_-receptor antagonist bicuculline. This proves the participation of GABA-ergic mechanisms in regulation of brain vessels' tone to realize cerebrovascular effects of studied compound.

As a conclusion, it should be noted that 5-hydroxyadamantane-2-on, an adamantane derivative, which is not a NMDA receptor antagonist, has significant cerebrovascular and neuroprotective activity in conditions of global and local brain ischemia which is manifested by enhancement of blood flow of ischemic brain, prevention of structural disturbances, and increase in survival rate of animals with global ischemia provoked by hypergravity loading. It can be supposed that GABA-ergic system of brain vasculature has a great value in cerebrovascular and neuroprotective activities of 5-hydroxyadamantane-2-on. Our results conform to the literature data as well as to the results of our own preview studies, according to which compounds enhancing GABA-ergic conduction, agonists and modulators of GABA_A_ receptors, inhibitors of GABA transaminase, GABA reuptake inhibitors, possess neuroprotective activity [6, 11–15].

## Figures and Tables

**Table 1:** The influence of 5-hydroxyadamantane-2-on (5-HA-2-on) on survival rate of animals in conditions of hypergravity loading.

	Control		5-HA-2-on		5-HA-2-on		5-HA-2-on		5-HA-2-on	
			50 mg/kg		100 mg/kg		150 mg/kg		200 mg/kg	
	*N*	%	*N*	%	*N*	%	*N*	%	*N*	%
Number of survived animals	2	20	2	20	8*	80	6	60	6	60
Number of dead animals	8	80	8	80	2	20	4	40	4	40

*P ≤ 0.05.
